# A Driving Warning System for Explosive Transport Vehicles Based on Object Detection Algorithm

**DOI:** 10.3390/s24196339

**Published:** 2024-09-30

**Authors:** Jinshan Sun, Ronghuan Zheng, Xuan Liu, Weitao Jiang, Mutian Jia

**Affiliations:** 1 State Key Laboratory of Precision Blasting, Jianghan University, Wuhan 430056, China; sunjinshan@cug.edu.cn; 2 Hubei Key Laboratory of Blasting Engineering, Jianghan University, Wuhan 430056, China; 3 School of Civil Engineering, Tianjin University, Tianjin 300350, China; 2021205249@tju.edu.cn (R.Z.); liuxuan_94@tju.edu.cn (X.L.); jwt@tju.edu.cn (W.J.)

**Keywords:** explosive transport vehicle, YOLOv4 object detection algorithm, cellular automata, intelligent recognition

## Abstract

Due to the flammable and explosive nature of explosives, there are significant potential hazards and risks during transportation. During the operation of explosive transport vehicles, there are often situations where the vehicles around them approach or change lanes abnormally, resulting in insufficient avoidance and collision, leading to serious consequences such as explosions and fires. Therefore, in response to the above issues, this article has developed an explosive transport vehicle driving warning system based on object detection algorithms. Consumer-level cameras are flexibly arranged around the vehicle body to monitor surrounding vehicles. Using the YOLOv4 object detection algorithm to identify and distance surrounding vehicles, using a game theory-based cellular automaton model to simulate the actual operation of vehicles, simulating the driver’s decision-making behavior when encountering other vehicles approaching or changing lanes abnormally during actual driving. The cellular automaton model was used to simulate two scenarios of explosive transport vehicles equipped with and without warning systems. The results show that when explosive transport vehicles encounter the above-mentioned dangerous situations, the warning system can timely issue warnings, remind drivers to make decisions, avoid risks, ensure the safety of vehicle operation, and verify the effectiveness of the warning system.

## 1. Introduction

Explosive transport vehicles are extremely dangerous carriers that typically carry highly flammable, explosive, or chemically hazardous substances. Explosives include various explosives, gunpowder, strong gases, and chemicals, which are highly susceptible to explosions or fires under improper external effects, resulting in huge casualties and property damage [1]. Although explosive transport vehicles usually adopt a series of safety measures, such as special design, isolation, and fire prevention measures, to minimize the losses caused by accidents, in extreme cases, these measures may fail [2]. Therefore, timely detection and warning of dangers during transportation are crucial to assist drivers in making correct decisions at critical moments [3].

Due to the flammable and explosive nature of the items carried by explosive transport vehicles, their vehicles themselves pose greater risks compared to other types of vehicles. Especially in urban centers or other areas with relatively dense crowds and buildings, the road and traffic conditions are usually complex. Explosive transport vehicles are prone to abnormal collisions and friction with pedestrians and vehicles when driving in these areas, which can easily lead to explosions, fires, and other dangers for explosive transport vehicles. Moreover, these places often have flammable and explosive hazards such as gas stations, liquefied petroleum gas storage and distribution stations, gas pipeline systems, and flour mills. Therefore, once an explosive transport vehicle explodes or catches fire in these areas, it usually causes extremely serious casualties and huge economic losses [4].

Due to the significant potential hazards of explosive transport vehicles during operation, ensuring their transportation safety has received increasing attention. Relevant algorithms for detecting and identifying surrounding vehicles during vehicle operation are also gradually being valued. At present, vehicle target detection algorithms are mainly divided into R-CNN series [5,6], YOLO series [7,8], and SSD algorithms [9,10]. The R-CNN series algorithms mainly use candidate region search and classify and locate candidate regions through deep convolutional neural networks (CNN); the YOLO series algorithms transform the object detection problem into a regression problem, achieving end-to-end object detection through a single CNN model; the SSD algorithm uses multiple anchor boxes of different sizes and aspect ratios to predict the category and position information of targets through feature maps at different levels, achieving fast detection. Scholars have also conducted extensive research and functional improvements on the above target detection algorithms. Zhu Yunfei et al. [11] proposed an improved Faster R-CNN vehicle recognition algorithm to solve the problem of false detection between vehicles with similar appearances and the impact of partial occlusion conditions on detection accuracy, making the model more sensitive to vehicle type recognition in complex scenes; Zhang Zhao et al. [12] proposed the deconvolution reverse feature fusion fast R-CNN algorithm by fusing feature pyramid network (FPN) and fast RCNN, which solved the problem of poor detection performance of fast R-CNN for long-distance small target vehicles; Zhou Mengguang [13] has made improvements to the YOLO series model to increase the detection accuracy of long-distance pedestrians and enhance the accuracy of the model; Yin Yuanqi et al. [14] proposed an improved object detection algorithm YOLO v4 ASC based on YOLO v4, which improves the problems of low detection accuracy and poor detection performance of small targets caused by occluded targets in vehicle object detection; Kangruijie [15] has improved the SSD algorithm to address the issues of small targets and vehicle missed detections caused by occlusion, thereby enhancing the detection accuracy of the algorithm; Yang Fan and Wu Shaobo [16] improved the anchor based on the SSD algorithm and added infrared thermal images to visible light data to optimize the model, effectively improving the accuracy of vehicle detection. These algorithms have been widely applied after targeted optimization based on their characteristics and different uses. For the explosive transport vehicle studied in this article, multiple vehicles usually appear around it during driving. The YOLOv4 object detection algorithm can effectively handle large-scale object detection tasks and has high processing efficiency for situations where multiple vehicles need to be detected simultaneously. This algorithm can balance speed and accuracy well, and it has a very fast processing speed while maintaining high detection accuracy, which makes it have significant advantages over other algorithms in real-time detection. In addition, the YOLOv4 algorithm is also compatible with different hardware and platforms, including CPUs and GPUs, which makes it have strong hardware adaptability and can be deployed in various environments.

**Figure 1:**
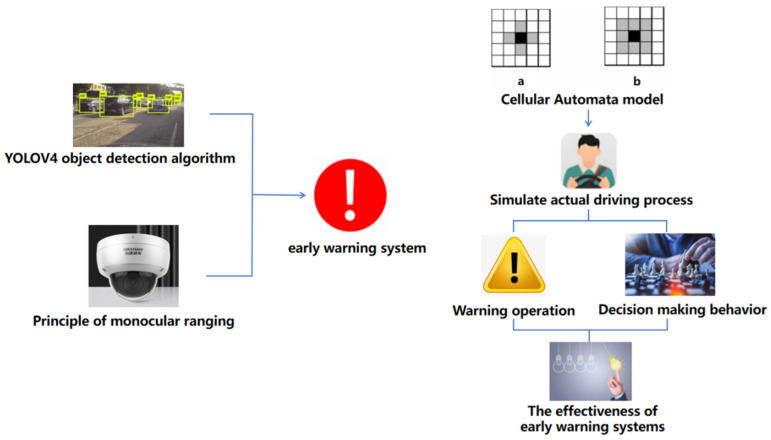
Framework diagram of research ideas.

Based on the analysis of existing research status, it can be seen that vehicle recognition target detection algorithms can, to some extent, achieve warning and avoidance of dangers during vehicle driving. However, there are still many problems in the practical application of this technology: (1) At present, there is a lack of specialized development scenarios for explosive transport vehicles, and universal object detection models lack guarantee of generalization; (2) intelligent driving systems equipped with this algorithm typically require a large amount of sensors, computing resources, and advanced software, which can lead to high costs and hinder the popularization and application of algorithms and related technologies; (3) for the intelligent driving system itself, it needs to handle a large amount of data and complex algorithms, which increases the complexity of the system and also increases the probability of system failure. To solve the above problems, the YOLOv4 object detection algorithm is planned to be used, and the model is trained accordingly. At the same time, monocular camera ranging technology is combined to measure the distance of objects through a single camera. Image processing and computer vision algorithms are used to analyze the size and position of objects in the image. Usually, principles such as size estimation, disparity method, feature extraction, and matching are used to infer the distance of objects. Flexible placement of consumer-grade cameras around the vehicle body to monitor surrounding vehicles in order to achieve identification and warning of abnormal approaches or lane changes of explosive transport vehicles during operation. In addition, this article also uses a cellular automaton model based on game theory to simulate real urban traffic flow and studies the decision-making behavior of drivers when encountering the above-mentioned dangerous situations during actual driving, verifying the effectiveness of the early warning system. The explosive transportation vehicle driving warning system based on the above object detection algorithm has promoted the application of the YOLOv4 object detection algorithm in vehicle recognition and warning. This study combines hardware layout to improve and optimize the algorithm, which has good practical application value. The specific research framework is shown in Figure 1.

Overall, due to the flammable and explosive nature of explosives, the existing protective measures for explosive transport vehicles make it generally difficult to ensure timely and effective protection in case of danger. It is more economical and reliable to take measures to prevent risks rather than focus on protection after a danger occurs. This article proposes a driving warning system based on the YOLOv4 object detection algorithm, which realizes the detection of abnormal approaching and lane changing situations of vehicles around explosive transport vehicles. It timely alerts drivers to take measures to avoid risks before danger occurs. This article also uses a cellular automaton model based on game theory to simulate actual driving scenarios and verify the effectiveness of this warning system. The results show that the warning system can effectively warn drivers of risks, remind drivers to take measures to avoid risks, and ensure driving safety. This warning system solves the problems of high hardware costs and complex algorithms in current intelligent driving technology and, to a certain extent, expands the application fields of consumer-grade cameras with lower technical requirements, providing broader application prospects for this type of camera in vehicle driving environment detection, intelligent recognition, and related fields.

Explanation for this study:

The camera parameters used in this study were taken from Hikvision.

The software used in this article is Python 3.7.3, with the following parameters:

scipy==1.2.1

numpy==1.17.0

matplotlib==3.1.2

opencv_python==4.1.2.30

torch==1.2.0

torchvision==0.4.0

tqdm==4.60.0

Pillow==8.2.0

h5py==2.10.0

Website: https://github.com/bubbliiiing/yolov4-pytorch/blob/master/requirements.txt, accessed on 8 June 2024.

## 2. Single Camera Layout Plan

### 2.1. Single Camera Ranging

**Figure 2:**
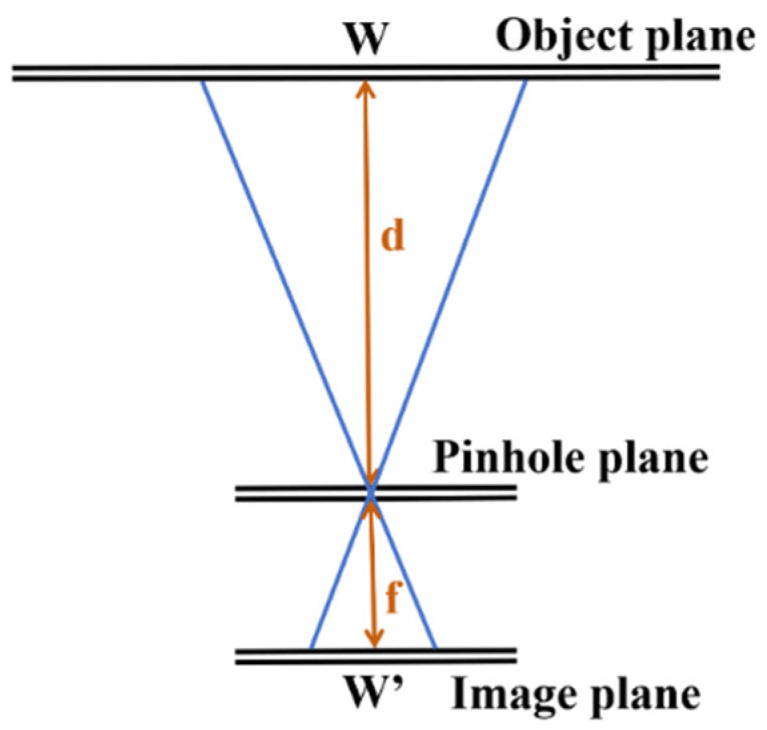
Schematic diagram of monocular camera ranging principle.

According to the principle of camera imaging, the size of an object in the camera image is proportional to its actual size, which is related to the focal length of the camera. Therefore, the principle of similar triangles can be used to calculate the distance between vehicles, achieving the function of monocular cameras to roughly measure the distance between vehicles. The principle is shown in Figure 2.

w is the actual size of the object, $w^{\prime }$ is the pixel size of the object on the imaging plane, *d* is the distance between the object and the camera pinhole plane, and *f* is the focal length. The relationship between the actual size and distance of an object can be obtained from the similarity of triangles:

(1) $$\frac{w}{w^{\prime }}=\frac{d}{f}$$

From this, the distance between the object and the camera can be obtained:

(2) $$d=\frac{w}{w^{\prime }}f$$

### 2.2. Camera Layout Plan

Explosives belong to dangerous goods, and their transportation should comply with relevant regulations for the transportation of dangerous goods. In terms of driving speed, according to Article 46 of Chapter 7 of China’s Measures for the Safety Management of Road Transport of Dangerous Goods, dangerous goods transport vehicles shall not exceed 80 km per hour on highways and 60 km per hour on other roads. If the speed indicated on road speed limit signs and markings is lower than the prescribed speed, the vehicle’s driving speed shall not exceed the speed indicated on the speed limit signs and markings. In terms of vehicle distance, Article 45 of the Implementation Regulations of the Road Traffic Safety Law of the People’s Republic of China stipulates that motor vehicles shall not exceed the speed indicated by speed limit signs and markings on the road. On roads without speed limit signs or markings, motor vehicles are not allowed to exceed the following maximum driving speeds: on roads without a centerline, 30 km per hour for urban roads and 40 km per hour for highways; a road with only one motor vehicle lane in the same direction has a speed of 50 km per hour for urban roads and 70 km per hour for highways. Article 80 stipulates that when a motor vehicle is driving on a highway at a speed exceeding 100 km per hour, it shall maintain a distance of more than 100 m from the preceding vehicle in the same lane. When the speed is below 100 km per hour, the distance from the preceding vehicle on the same lane may be appropriately shortened, but the minimum distance shall not be less than 50 m. According to relevant regulatory requirements, combined with the general situation of explosive transport vehicles during operation and the ranging accuracy error of consumer-level cameras, the safe distance of the vehicle is set to 60 m between the front and rear vehicles, and 1.5 m between the left and right vehicles, as the warning conditions for the warning system. When the distance between vehicles is less than the safe distance mentioned above, the warning system will prompt an alarm if the surrounding vehicles approach or change lanes abnormally.

Focal length refers to the distance from the center of the lens to the focal point of light concentration when parallel light is incident, and it also refers to the distance between the optical center point of the lens and the CCD/CMOS photosensitive element of the camera in a surveillance camera. The larger the focal length of the camera, the smaller the range of view that can be seen, but the clearer the details; on the contrary, the smaller the focal length, the wider the range of viewing angles that can be seen, but the more blurry the details.

The driving warning system studied in this article mainly detects front, rear, and side vehicles, observing whether they have abnormal lane-changing behavior. Therefore, based on the performance characteristics and usage requirements of the camera itself, the selected camera and its related parameters are shown in Table 1.

**Figure 3:**
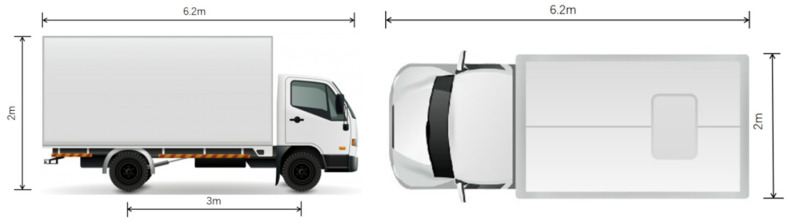
Dimensions of freight cars.

As shown in Figure 3, a 3.5 ton box truck (length 6.2 m × width 2.0 m × height 2 m) is selected as the camera layout object, with a wheelbase of 3 m. It is recommended to set a monitoring blind spot length of about 1.5 m between the left and right vehicles and a horizontal field of view angle of 90°. By substituting the selected camera parameters into Equation (2), the distance between the object and the camera can be obtained.

**Figure 4:**
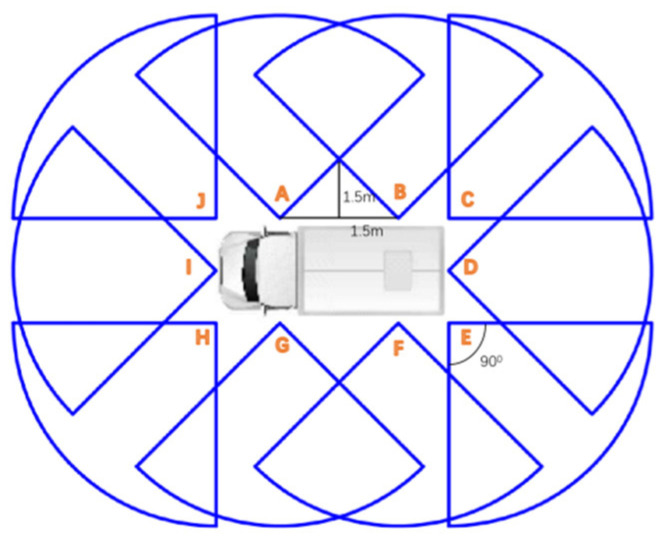
Camera layout.

Based on the above analysis, it can be concluded that the camera layout scheme is shown in Figure 4, with a total of 10 cameras, labeled as A to J.

**Figure 5:**
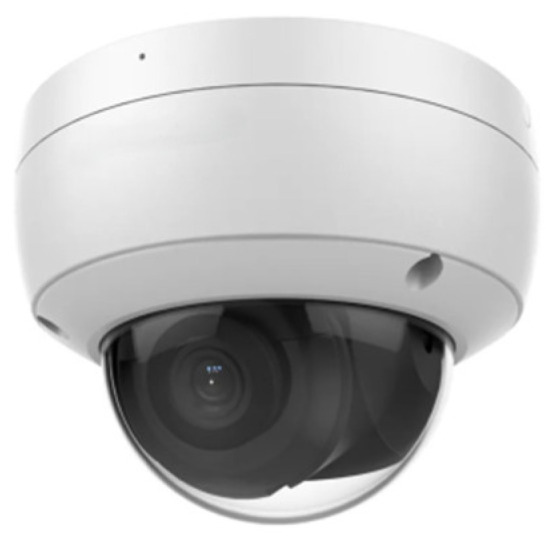
Camera appearance.

Please refer to Figure 5 for the appearance of the camera.

Note: All cameras are placed at a height of 1.5 m above the ground on the truck.

## 3. Implementation Steps of YOLO V4 Target Monitoring Algorithm

### 3.1. Recognition Principle

YOLOv4 is inherited from the YOLO series by Alexey Bochkovskiy, and has been improved on the basis of YOLOV3. While maintaining performance comparable to the EfficientDet network, YOLOv4’s inference speed is about twice that of EfficientDet. Compared with the previous generation, YOLOV3, its algorithm has significantly improved average accuracy and FPS [18].

**Figure 6:**
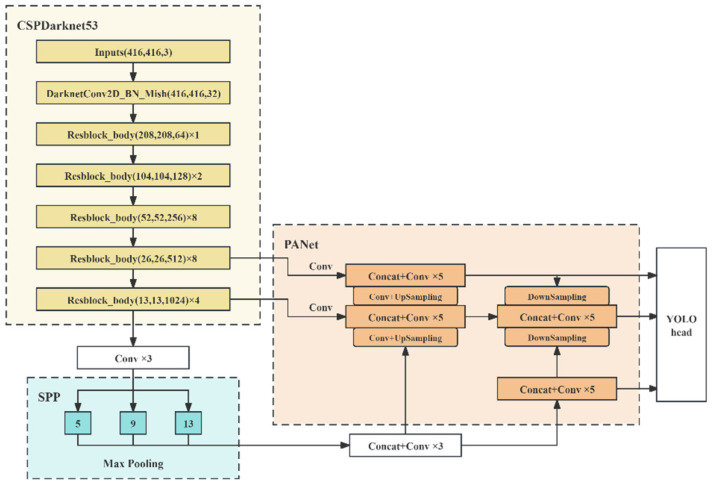
YOLOv4 network structure [19].

The network structure of YOLOv4 is shown in Figure 6.

YOLOv4 is the fourth version of the YOLO series algorithm, which builds a powerful framework and achieves efficient real-time object detection through deep learning technology. Compared with previous versions, YOLOv4 has made significant progress in speed and accuracy, aiming to achieve efficient real-time object detection and localization, enabling computers to understand and perceive the surrounding environment. YOLOv4 is a single-stage detector that performs object detection and localization simultaneously in a forward pass, without the need for multiple stages or models. In terms of network architecture, YOLOv4’s network architecture is based on deep convolutional neural networks (CNNs), which typically use very deep neural networks such as Darknet or CSPDarknet to extract image features and perform object detection. YOLOv4 uses anchor boxes to predict targets of different sizes and aspect ratios, which helps improve detection capabilities for various target sizes. In terms of achieving multi-scale detection and category detection, YOLOv4 can simultaneously capture objects of different sizes in the image by detecting them at multiple scales. It processes multiple scales through feature pyramids and performs object detection on each scale. Not only can it detect the position of objects, but it can also predict the category of each detected object, which means it can recognize multiple targets of different categories. In terms of post-processing, YOLOv4 performs post-processing steps such as non-maximum suppression (NMS) to remove overlapping detection bounding boxes and ensure that each target is only detected once. The performance of YOLOv4 depends on a large and diverse training dataset, which includes objects of different categories and sizes. Overall, YOLOv4 is a fast, accurate, and efficient object detection algorithm suitable for various real-time detection situations.

### 3.2. Model Training

#### 3.2.1. Load Dataset

**Figure 7:**
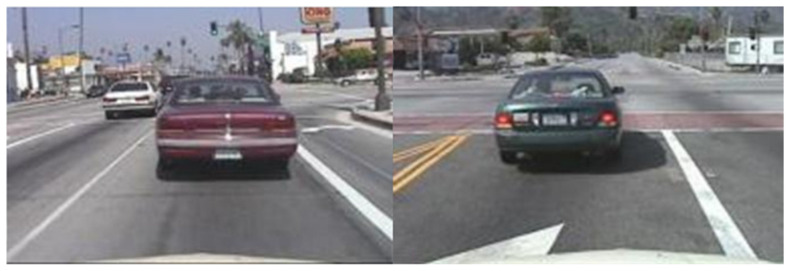
Example of vehicle data images.

Use a dataset containing 1000 vehicle images and divide it into three parts: training set, validation set, and test set. Select 60% of the data for training, 10% for validation, and the remaining for testing trained detectors. Considering the complexity of YOLOv4 in recognizing vehicles in practical situations, the vehicle images used for training include various situations and scenes, including vehicle images taken in rainy and snowy weather, vehicle images taken in smoke covered conditions, vehicle images taken in strong light exposure, etc. Ensure that the dataset contains diverse scenarios and weather conditions to improve the recognition performance of the model, enhance its robustness and generalization ability. An example of a vehicle data image is shown in Figure 7.

**Figure 8:**
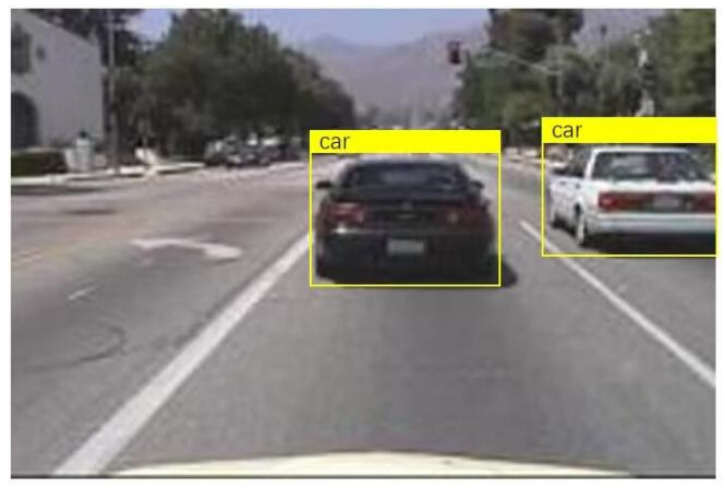
Training images and box labels.

To better observe the progress, one can choose to display one of the training images and box labels, as shown in Figure 8.

#### 3.2.2. Perform Data Augmentation

**Figure 9:**
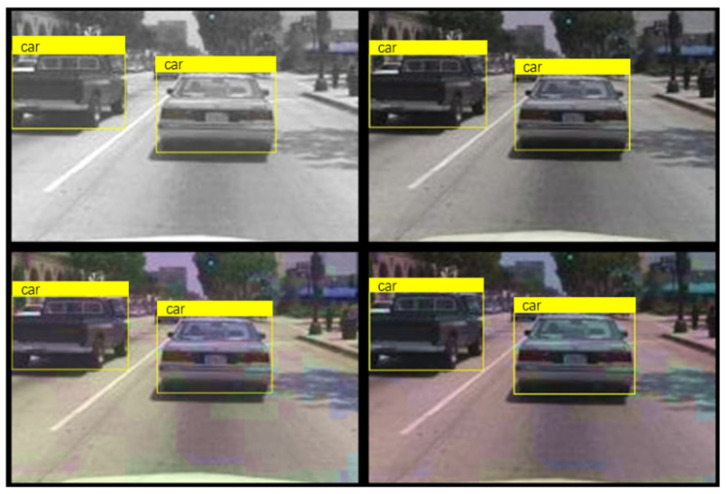
Enhanced training dataset.

To improve the accuracy of training, it is necessary to perform data augmentation operations. Firstly, apply custom data augmentation to the training data, and then expand the color jitter enhancement, random horizontal flipping, and random scaling by 10% in the HSV space to the input data. At this point, it should be noted that data augmentation is not applicable to testing and validation data. Ideally, testing and validation data should represent the original data and not be modified for an unbiased evaluation. Then read and display samples of enhanced training data. The enhancement operation of the training dataset is shown in Figure 9.

**Figure 10:**
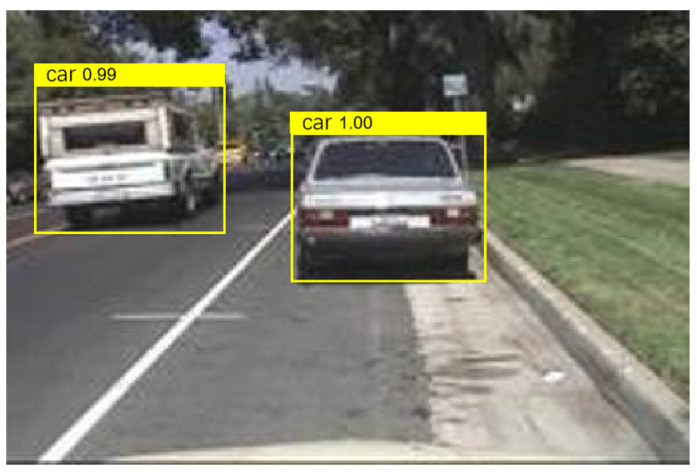
The results of training.

After the training is completed, use the test dataset to evaluate the trained detectors and view the training results. From Figure 10, it can be seen that the detector is already able to identify the vehicle well. When YOLOV4 recognizes a target in the image, the 0.98 displayed on the label indicates the confidence level of the target. Confidence is an indicator used in object detection algorithms to represent the reliability of detection results. It reflects the degree of certainty of the model towards the detected target. In YOLOV4, the calculation of confidence is usually based on factors such as the degree of feature matching of the target, the accuracy of the bounding box, and the prediction probability of the model. A higher confidence level indicates that the model is more certain in detecting the target, while a lower confidence level indicates that there is some uncertainty in the model’s detection of the target. Generally speaking, the confidence threshold can be adjusted according to specific application requirements. A higher threshold can improve the accuracy of detection, but it may lead to some targets being missed; a lower threshold can increase the recall rate of detection, but it may also introduce some false positives. In practical applications, it is necessary to balance accuracy and recall rate according to specific situations and choose an appropriate confidence threshold to meet the requirements. At the same time, other indicators such as accuracy, recall, and F1 score can be combined to comprehensively evaluate the performance of the model.

**Figure 11:**
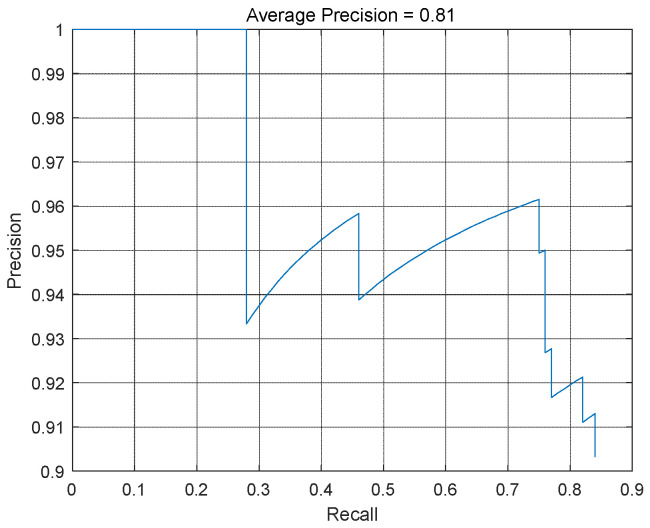
PR curve.

**Figure 12:**
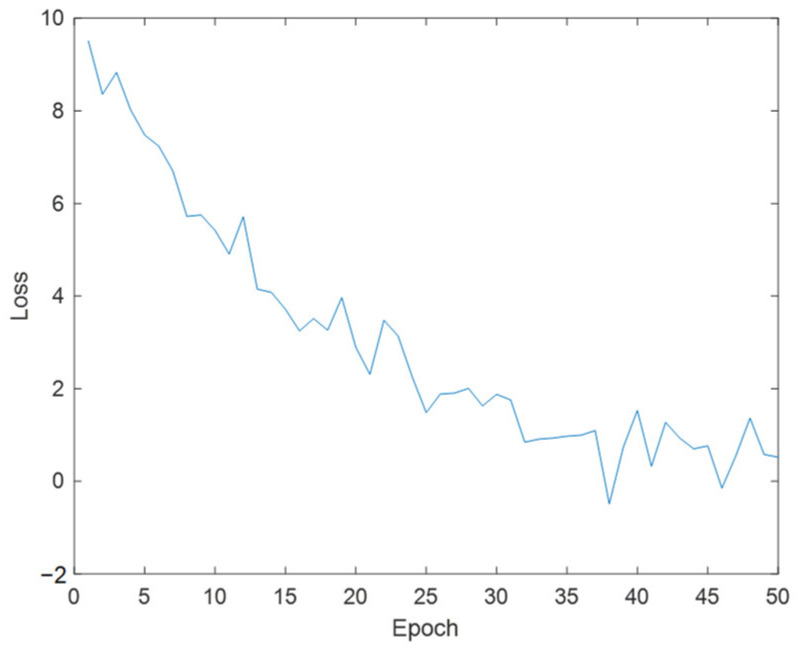
Loss function curve.

Evaluate the performance of trained detectors using the average accuracy metric. The average accuracy provides a number that includes the detector’s ability to classify correctly (accuracy) and the detector’s ability to find all relevant objects (recall rate). The precision recall (PR) curve highlights the accuracy of the detector at different recall levels. At all recall levels, the ideal accuracy is 1. Using more data can help improve average accuracy, but it may require more training time. Finally, draw the PR curve, as shown in Figure 11. The loss function curve is shown in Figure 12.

It should be noted that the intermittent increase and decrease in the vertical axis (i.e., accuracy) in the PR curve drawn after YOLOv4 training may be due to the following reasons: (1) threshold adjustment. The drawing of PR curves usually involves different threshold settings. By changing the threshold, the classification criteria for positive and negative examples can be determined. When the threshold is adjusted, the number of samples judged as positive will change, thereby affecting the accuracy of the calculation. If the proportion of true positive cases in the newly determined positive cases is high, the accuracy will increase; on the contrary, if a large number of negative examples in the newly determined positive examples are misjudged as positive examples, the accuracy will decrease. (2) Sample distribution and characteristics: The distribution and characteristics of samples can also affect accuracy. Different samples may have different levels of difficulty and characteristics, resulting in fluctuations in classification accuracy at different thresholds. Some samples may be easier to classify correctly, while others may be more challenging, leading to intermittent variations in accuracy. (3) Model performance and characteristics: The performance and characteristics of the YOLOv4 model itself can also affect changes in accuracy. The model may have better classification ability for certain types of samples, while it may perform poorly for other types of samples. This uneven performance may lead to intermittent changes in accuracy during the threshold adjustment process.

The loss function is a function used to measure the degree of difference between the predicted results of a model and the true values. The loss function curve is a curve that records the loss function values calculated after each iteration or epoch during the training process of the model and plots these values. During model training, the parameters of the model are continuously adjusted through optimization algorithms to minimize the value of the loss function. The loss function curve can help us understand the training progress of the model. Normally, as training progresses, the loss function value gradually decreases, indicating that the model is gradually learning and improving. If the curve shows a significant downward trend and eventually stabilizes, it indicates that the model training effect is good. If the curve fluctuates significantly or cannot converge, it may be necessary to adjust the training parameters or model structure. The loss function curve trained by this model shows that as the training process progresses, the curve gradually decreases and eventually stabilizes.

After training, the model can accurately recognize surrounding vehicles, capture real-time vehicle information, and monitor vehicle distance. When the distance between the left and right vehicles is less than or equal to 1.5 m or the distance between the front and rear vehicles is less than 60 m, it is judged as an abnormal approach of the surrounding vehicles, and a warning is issued. Due to the fact that the false alarm cost of monitoring targets around explosive transport vehicles is lower than the false alarm cost, combined with the actual situation of this study, we have chosen to set a confidence threshold of 0.4.

YOLO receives real-time video image information transmitted by the camera, first identifies the vehicle, and then obtains the pixel size *w*′ of the anchor frame of the identified vehicle on the imaging plane. For front and rear vehicles, the program can calculate the distance between the vehicle and the camera based on the width of the vehicle. Given the width of the vehicle, the focal length of the camera, and the pixel width of the vehicle on the imaging plane (i.e., anchor frame width), the distance between the vehicle and the camera (i.e., vehicle distance) can be calculated. This also applies to the distance measurements of left and right vehicles. Given the length of the vehicle, the distance can be calculated. In the camera used in this study, the focal length *f* was set to 8 mm.

According to Formula (2), we can draw the following conclusion:

Assuming the length and width of the vehicle are 4000 mm and 2000 mm, respectively, the distance between vehicles is:

(3) $$d_{1}=\frac{4000}{w^{\prime }}\times 8$$

(4) $$d_{2}=\frac{2000}{w^{\prime }}\times 8$$

**Figure 13:**
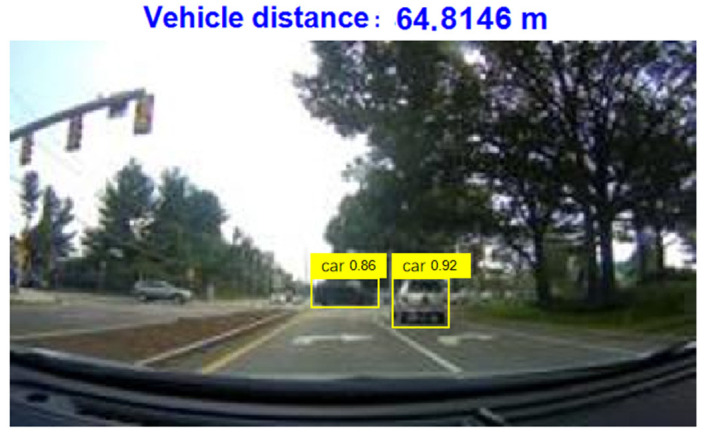
Capturing vehicle image information (safe vehicle distance).

Under safe vehicle distance conditions, the captured vehicle image information is shown in Figure 13.

**Figure 14:**
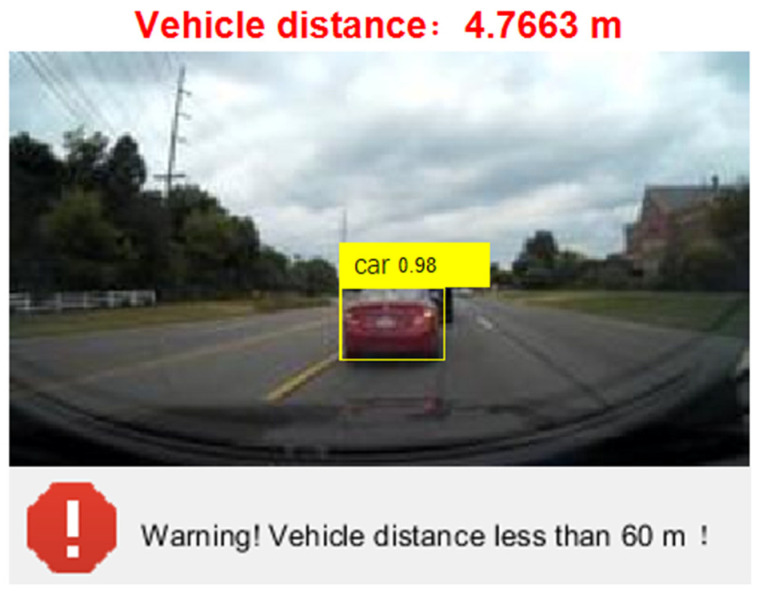
Capture vehicle image information (when the current rear distance is less than 60 m or the left and right distance is less than 1.5 m).

The program monitors the distance between the front and rear vehicles and the distance between the left and right vehicles in real time and sets the program to automatically prompt and alarm when it detects that the distance between the front and rear vehicles is less than 60 m or the distance between the left and right vehicles is less than 1.5 m. When the distance behind is less than 60 m or the distance between left and right is less than 1.5 m, capture the vehicle image information as shown in Figure 14.

## 4. Simulation of Vehicle Operation Using Cellular Automata Based on Game Theory

The simulation of vehicle operation using cellular automata based on game theory is a complex system modeling method used to simulate the behavior and traffic flow of vehicles in road networks. This model combines the principles of cellular automata and game theory to understand and simulate the decision-making and interaction processes of vehicles on the road. Cellular automata [20] is a discrete model that divides space into discrete cells, each of which can be in a different state. In road traffic simulation, each cell typically represents a discrete location on the road, which can be a vehicle or a blank section of the road. The time of the model is also discrete, with each time step simulating the movement and decision making of vehicles on the road. Each vehicle is modeled as an independent agent in cellular automata, which can make decisions based on surrounding conditions. Vehicle decisions can include behaviors such as acceleration, deceleration, and lane changing, which are usually influenced by traffic rules and game strategies [21]. Game theory is used to describe the interaction and competition between vehicles, as well as their optimal decisions under different strategies. Vehicles can be seen as players participating in the game, and they choose strategies based on their own goals and constraints [22]. The rules of cellular automata describe the interactions between vehicles, including how to change speed, how to change lanes, etc. These rules are based on strategies and decisions in game theory, as well as the road conditions around the vehicles. During the simulation process, the model simulates the behavior of vehicles and the evolution of road conditions at each discrete time step. Vehicles make decisions based on game strategies, and their position and speed will change over time. This game theory-based cellular automaton model helps researchers simulate and understand the interaction behavior between vehicles and the formation and evolution processes of traffic flow on the road. It can be used to evaluate the effectiveness of different traffic management strategies, study the causes of traffic congestion, and optimize road network design [23]. Such models can play a crucial role in transportation planning, the development of intelligent transportation systems, and urban traffic management. This study is based on game theory and cellular automata principles and uses a stochastic traffic flow simulation program to establish an urban traffic flow model. Simulate the program warning situation and decision-making process of drivers driving explosive transport vehicles equipped with the above warning systems on actual roads when encountering abnormal approaches or lane changes from surrounding vehicles; verify the effectiveness of the warning program, that is, whether taking measures when the driver receives an alarm from the warning program can effectively avoid accidents.

### 4.1. Enter Initial Parameters

Firstly, define the cellular automaton model, which includes the length of the simulated road segment, simulation duration, and number of lanes. Set the number of lanes to 3, with each lane having a length of 200 cells and a simulation step size of 1000. Initialize the lane change counter, the safe distance following the vehicle, the simulation time step, the structure of the driving vehicle, the structure of the exiting vehicle, the vehicle number, and the vehicle number counter. Set the vehicle acceleration, lane change probability, and deceleration probability. Please refer to Table 2 for detailed parameters.

During the operation of the program, some vehicles will be randomly designated as explosive transport vehicles, while the rest are normal vehicles. As this study mainly focuses on the safety of explosive transport vehicles, there are not too many requirements for the safe distance of other vehicles; that is, no collision is required. When other vehicles approach or change lanes abnormally around the explosive transport vehicle, the vehicle is marked in red, and the program prompts a warning.

The cellular automaton model used in this study combines NaSch car-following rules with custom lane-changing rules. The NaSch (Nagel Schreckenberg) model is a microscopic traffic model proposed by German physicists Kai Nagel and Stephen Schreckenberg in 1992 for simulating traffic flow. The basic assumption is that the vehicle is traveling on a one-dimensional road, and each vehicle can only move in integer positions. The evolution of the model is based on the following rules:

1. Acceleration rule: If the current speed of the vehicle is less than the maximum speed limit, it can accelerate. Usually, vehicles can accelerate by 1 unit per time step until reaching maximum speed.
2. Deceleration rule: If the distance ahead of the vehicle is less than its current speed, it must slow down to avoid collisions. This is to simulate the safe distance between vehicles.
3. Random deceleration rule: Introduce a random factor to simulate random deceleration in traffic flow. This makes the model closer to the complexity of actual traffic flow.
4. Movement rule: Vehicles move at their speed, that is, at each time step, by a distance equal to their current speed. The simulation process of the NaSch model is carried out by iteratively applying these rules to simulate the movement of vehicles on the road. The following rule considers the following behavior between vehicles, while the lane changing rule takes into account factors such as speed difference, distance ahead, safety distance, and emergency lane changing.

Note: $D_{safe}$ in Table 2 refers to explosive transport vehicles.

In this simulation, the number of lanes is set to 3, and the length of each cell is set to 6 m. The maximum speed of the vehicle is three cell lengths per time step (one time step is one second), and the safe distance between the front and rear of the explosive transport vehicle is 60 m, which is 10 cell lengths. The probability of random deceleration of the vehicle is set to 0.3, the probability of lane change is set to 0.5, the length of the vehicle is set to 1 cell length, and the total number of time steps in the entire simulation process is 1000.

### 4.2. Following Rules

The following rules describe how a vehicle adjusts its speed based on the position and speed of the preceding vehicle during driving. The following are detailed car-following rules:

1. Calculate the distance from the preceding vehicle:

For each vehicle *i*, calculate the distance between it and the preceding vehicle *i* + 1. Namely:

(5) $$d_{front}=x_{i+1}-x_{i}-l_{i}$$

Among them, $x_{i}$ is the current position of vehicle *i*, $l_{i}$ is the length of vehicle *i*.

2. Deceleration rules:

If $d_{front}$ < $v_{i}$, if the distance between vehicles is less than the speed of vehicle *i*, the vehicles will slow down to avoid collision. The degree of deceleration can be adjusted according to specific circumstances.

The speed $v_{i}$ of vehicle *i* is updated to:

(6) $$v_{i}=min(d_{front},v_{i})$$

3. Acceleration rules:

If $d_{front}>v_{i}$, if the distance between vehicles is greater than the speed of vehicle *i*, the vehicles can accelerate, but there is a certain limit to acceleration. The degree of acceleration can be adjusted according to specific circumstances. If the distance between vehicles is greater than the speed of vehicle *i*, the vehicles can accelerate, but there is a certain limit to acceleration. The degree of acceleration can be adjusted according to specific circumstances.

The speed $v_{i}$ of vehicle *i* is updated to:

(7) $$v_{i}=min(v_{max},v_{i}+1)$$

4. Maintain rules:

If $d_{front}=v_{i}$, if the distance between vehicles is equal to the speed of vehicle *i*, then the vehicles maintain their current speed.

5. Random deceleration rule:

With a certain probability, the vehicle decelerates randomly at a unit speed. This simulates the random behavior of the driver, increasing the randomness of the model.

The speed $v_{i}$ of vehicle *i* is updated to:

(8) $$v_{i}=max(0,v_{i}-1)$$

6. Mobile Rules

The vehicle moves at its current speed, i.e.,

(9) $$x_{i+1}=x_{i}+v_{i}$$

### 4.3. Lane Changing Rules

The following are the lane-changing rules adopted by the cellular automaton model, mainly based on several aspects. (1) Speed difference: If the speed of the vehicle in the current lane is slow while the speed of the vehicle in the other lane is fast, the vehicle tends to change lanes towards the faster lane. (2) Density difference: If the current lane where the vehicle is located is more crowded, but there are fewer vehicles in the other lane, the vehicle tends to change lanes towards the lane with lower density. (3) Forward distance: If the vehicle is currently in a lane with a closer distance ahead but a farther distance ahead in another lane, the vehicle tends to change lanes towards the lane with a farther distance ahead. (4) Emergency lane change: When vehicles on adjacent lanes make an emergency lane change, consider four situations, as shown in the following figure. (5) Safety distance: If the safety distance between the vehicle and other surrounding vehicles is too small, the vehicle may choose to change lanes.

**Figure 15:**
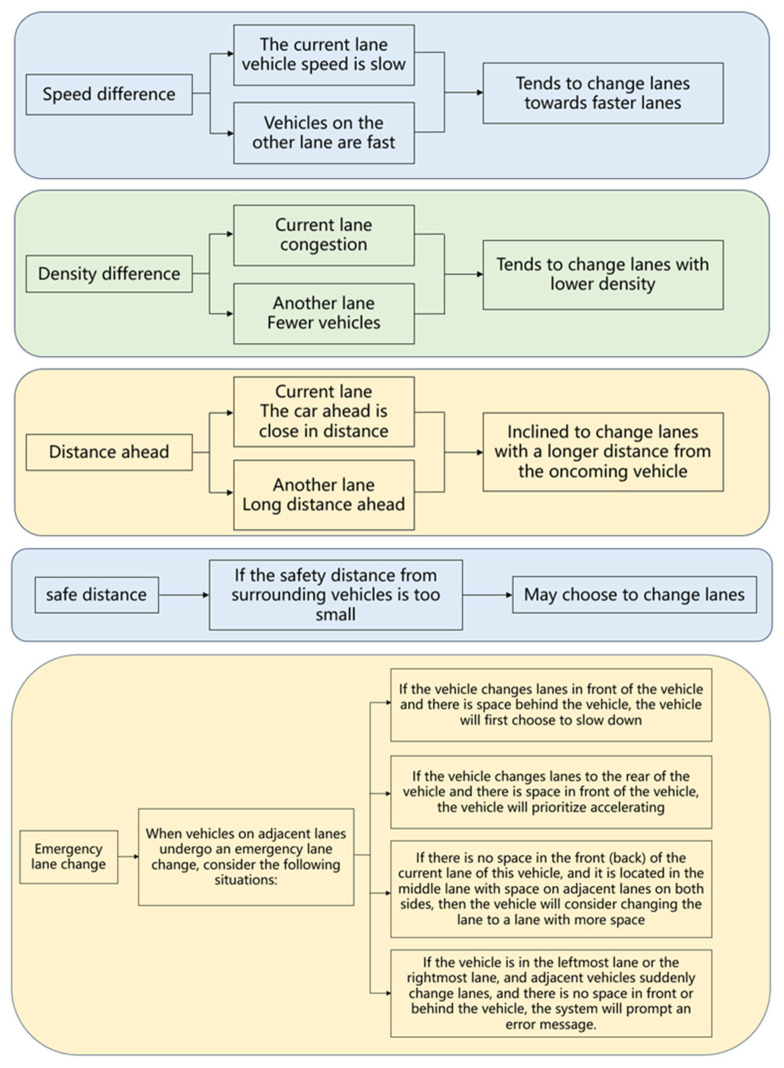
Lane-changing rules.

Please refer to Figure 15 for specific lane-changing rules.

### 4.4. Simulation Steps

**Figure 16:**
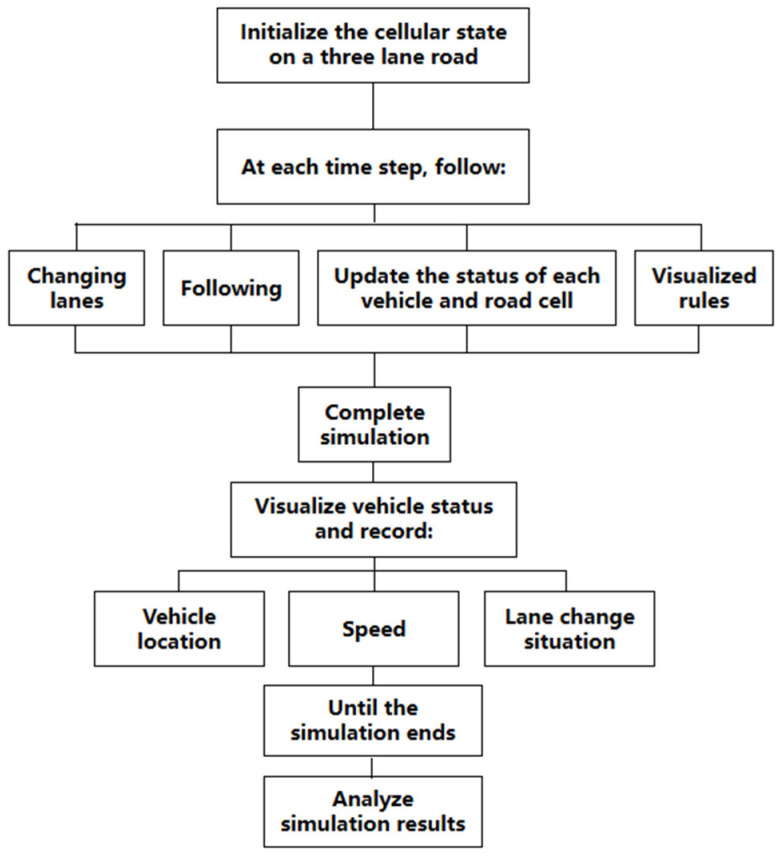
Flow chart of simulation steps for cellular automata.

Firstly, initialize the cellular states on three-lane roads, following the following settings at each time step: Changing lanes, following, update the status of each vehicle and road cell, visualized rules. Finally, complete the simulation, visualize the vehicle status and record the vehicle’s position, speed, and lane changes until the simulation is complete, and analyze the simulation results. The overall steps are shown in Figure 16:

### 4.5. Simulation Result

By running the simulation program, the simulation results are shown in the following figures.

**Figure 17:**
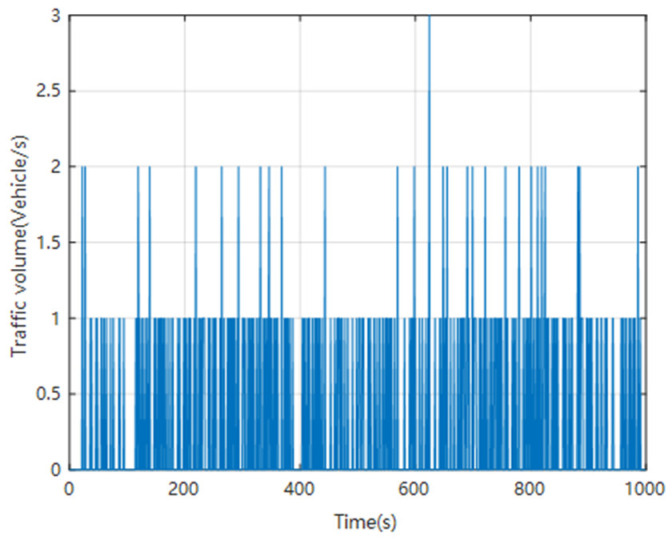
Traffic flow statistics.

Each time step is one second. From the beginning of the simulation, the traffic flow is counted at each time step, and the number of vehicles entering the lane from the leftmost side is counted at each time step. Due to the simulation being conducted step by step in time, only three situations will occur at the entrance of the lane: one vehicle entering one of the three lanes in this time step, two vehicles entering two lanes in each of the three lanes in this time step, and three vehicles entering each of the three lanes in this time step. Count a total of 1000 time steps and draw an image, as shown in Figure 17.

**Figure 18:**
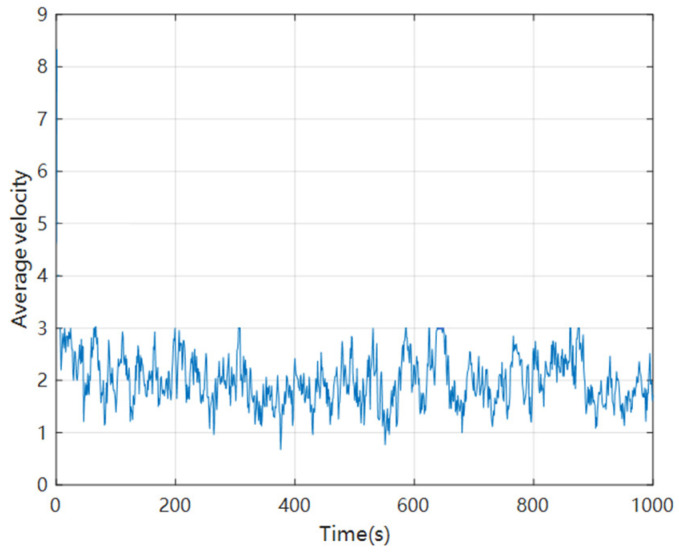
Statistical chart of average vehicle speed.

Calculate the average speed of all vehicles in these three lanes at each time step, that is, how many cell lengths the vehicles have moved at each time step, so the vertical axis has no units added, and draw a chart as shown in Figure 18.

**Figure 19:**
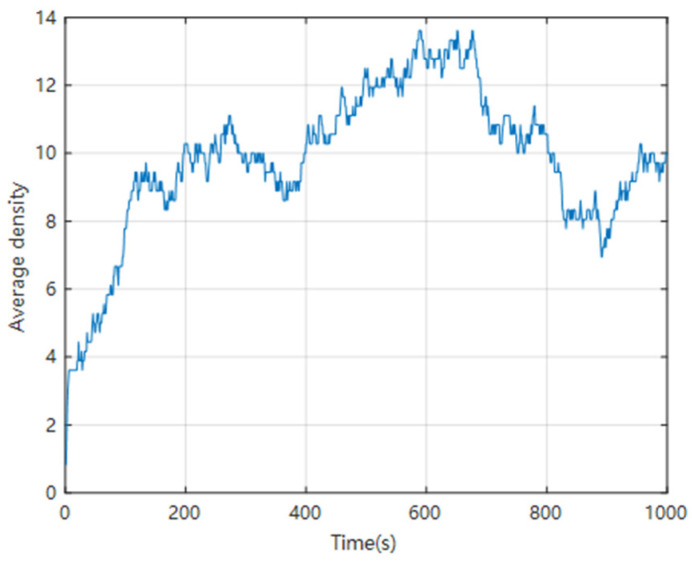
Statistical chart of average vehicle density.

Calculate the average density of vehicles in these three lanes at each time step and draw a chart; the vertical axis represents the number of vehicles, as shown in Figure 19.

From Figure 17, Figure 18 and Figure 19, it can be seen that throughout the simulation process, the average speed of the vehicle always fluctuates within a certain range, but does not exceed the specified maximum speed. The average density of vehicles initially gradually increases and then fluctuates within a certain range. It can be seen that the simulation results of this model are relatively reasonable.

**Figure 20:**
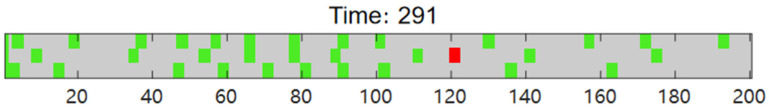
Simulation process of cellular automata (time step 291).

**Figure 21:**
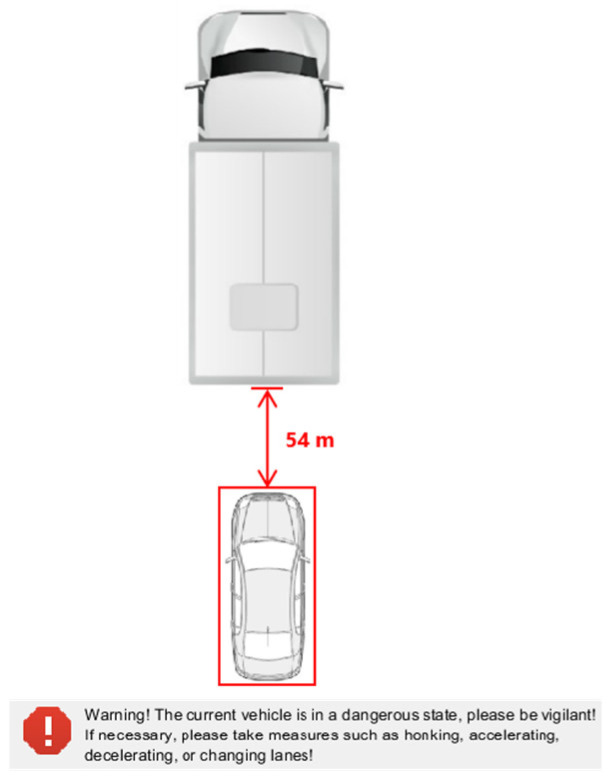
Program warning interface.

The cellular automaton simulates the actual driving process of the vehicle, as shown in Figure 20. Each block represents a vehicle traveling from left to right. The formation of vehicle traffic flow can be observed from the chart. The green square represents a normal vehicle, while the red square indicates that vehicles around the vehicle are approaching or changing lanes abnormally, indicating that the vehicle is in a dangerous state. This program can monitor vehicles driving in the lane in real time and issue warnings when explosive transport vehicles encounter the above-mentioned emergency situations, while simulating the driver’s decision-making process when encountering such dangerous situations. Figure 20 shows the road traffic conditions when the simulation step size is 291. Figure 21 shows the warning interface of the program when other vehicles approach or change lanes abnormally around the explosive transport vehicle.

**Figure 22:**
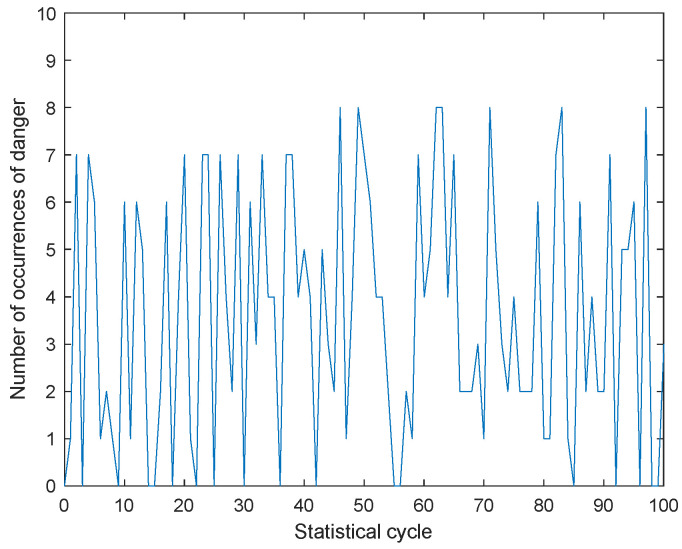
Collision statistics without warning system.

**Figure 23:**
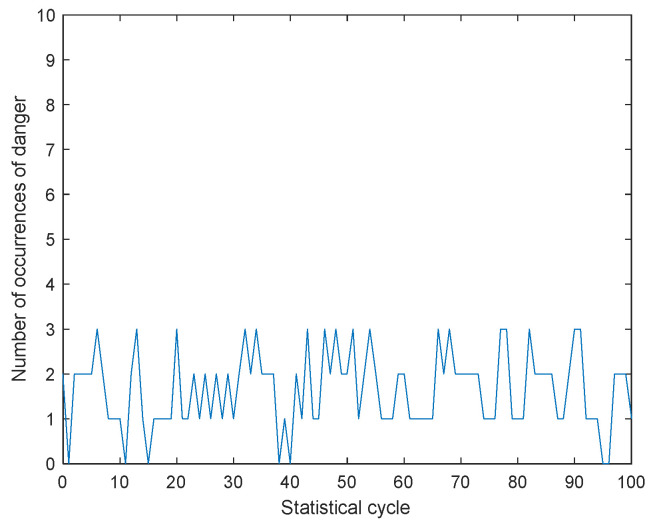
Collision statistics equipped with warning system.

In order to verify the effectiveness of the warning system during the actual driving process of explosive transport vehicles and whether the reactions made by the drivers of explosive transport vehicles can truly reduce the probability of vehicle collisions, we conducted two simulations using a cellular automaton model. Simulation regulation: When the distance between the explosive transport vehicle and surrounding vehicles is less than 1 m, it is considered a collision, which means an accident has occurred. The first simulation shows that explosive transport vehicles are not equipped with warning systems. During the simulation process, some vehicles are randomly designated as explosive transport vehicles, and their collision situations are monitored in real time. Every ten time steps, the number of collisions during this period is counted. The second simulated explosive transport vehicle is equipped with a warning system, and the driver can take timely measures to avoid risks after receiving the warning signal. Similarly, collision detection and calculation of collision frequency are also carried out. Comparing the two simulation results, it was found that in the absence of a warning system, collisions can occur up to 7 to 8 times every ten time steps. But with the equipped warning system, the average number of collisions occurring every ten time steps has decreased to 2 to 3, indicating that this warning system can significantly help explosive transport vehicle drivers avoid risks and ensure driving safety. The collision results of the two simulations are shown in Figure 22 and Figure 23.

## 5. Conclusions

This article proposes a driving warning system for explosive transport vehicles based on object detection algorithms and verifies the effectiveness of this system using a cellular automaton model:

- According to relevant road traffic regulations and selected consumer-level camera parameters, a camera layout plan for explosive transport vehicles has been designed to ensure compliance with the detection requirements of the surrounding environment during operation.
- This system includes recognition and distance measurement functions for surrounding vehicles, which are achieved through the YOLOv4 object detection algorithm. Then, using the principle of monocular camera distance measurement, the distance between vehicles is calculated based on the triangle similarity principle and the vehicle width and wheelbase. Based on the YOLOv4 code, improvements are made to achieve distance measurement. Promptly detect and warn drivers of abnormal approaches or lane changes in surrounding vehicles to prompt them to take appropriate measures.
- Using a cellular automaton model based on game theory, simulate the actual operating scenario of the vehicle, simulate the warning operation of the program, and the decision-making behavior of the driver when encountering dangerous situations while driving explosive transport vehicles. The results indicate that the model can effectively simulate real road traffic conditions and provide timely warning functions when explosive transport vehicles encounter abnormal approaches and lane changes from surrounding vehicles. At the same time, it can effectively simulate the decision-making process of the driver when encountering the above-mentioned dangerous situations. After the program warning, the driver can take timely acceleration, deceleration, or lane change measures, avoiding accidents and proving the effectiveness of the warning program.
- Research shortcomings and prospects: Currently, the testing of this study is only based on simulation and has not yet been deployed to hardware. Subsequent research will attempt to deploy it on real explosive transport vehicles and create a warning display screen for drivers to watch. Data will be collected in real-time situations to further verify the effectiveness and feasibility of the system.

## 6. Discussion

The YOLOv4 object detection algorithm in this article may have overfitting issues due to the characteristics of the model itself. We have used the following methods to avoid this problem as much as possible. (1) Increase data volume: We used more training data to reduce overfitting of the model to limited data and expanded the dataset through data augmentation techniques such as random rotation, scaling, flipping, etc. (2) Adjusting the model structure: We appropriately reduced the complexity of the model and the number of layers and neurons to avoid overfitting the training data. (3) Early stop method: During the training process, we monitor the performance indicators on the validation set. If the performance of the validation set does not improve after several consecutive epochs, we stop training in advance to avoid overfitting the model. (4) Data preprocessing: We preprocessed the data and standardized it, making it easier for the model to learn the features of the data and reducing the risk of overfitting. (5) Multi model fusion: We combine multiple different models for prediction, reducing the risk of overfitting for a single model and improving the generalization ability of the model. (6) Monitoring the training process: We closely monitor the indicators during the training process, such as loss function, accuracy, etc., promptly detect signs of overfitting, and take corresponding measures.

Overall, the system is based on consumer grade cameras, the YOLOv4 object detection algorithm, and cellular automata model, achieving intelligent detection of abnormal approaching and lane-changing situations of surrounding vehicles during the operation of explosive transport vehicles. It provides a guarantee for safe driving of vehicles, solves the problem of high hardware costs and complex algorithms for vehicle recognition in current intelligent driving technology, and expands the application field of consumer grade cameras with lower technical requirements to a certain extent, making it have broader application prospects in vehicle driving environment monitoring and related fields. We will continue to improve the functionality of the early warning system in subsequent research, constantly seeking simulation systems that are as close to the actual driving environment as possible to verify the practicality and effectiveness of the early warning system. At the same time, we will conduct experiments for on-site testing and ultimately apply the system to practice, ensuring the safe driving of explosive transport vehicles.

## Figures and Tables

**Table 1:** Camera parameter information [17].

Functional Requirement	Camera Model	Focal Length	Horizontal Field of View Angle	Irradiation Distance
Single camera rough measurement of front and rear vehicle distance	Hikvision 4 million infrared hemisphere 3146FWD-I camera	2.8–8 mm	37.5–98.2°	60 m

**Table 2:** Parameter table for cellular automata setting.

Number of Road Lanes	Cell Length	$\mathrm{Maximum}\;\mathrm{Vehicle}\;\mathrm{Speed}\;V_{max}$	$\mathrm{Minimum}\;\mathrm{Distance}\;\mathrm{between}\;\mathrm{Vehicles}\;D_{safe}$
3	6 m	3	10
$\mathrm{Random}\;\mathrm{deceleration}\;\mathrm{probability}\;P_{0}$	$\mathrm{Lane}\;\mathrm{changing}\;\mathrm{probability}\;P_{change}$	$\mathrm{Vehicle}\;\mathrm{length}\;l_{i}$	Total time *T*
0.3	0.5	1	1000

## Data Availability

The data presented in this study are available on request from the corresponding author on reasonable request.

## References

[1] Feng Y. (2022). Research on Intelligent Identification of Explosive Transport Vehicles and Fire Accidents. Master’s Thesis.

[2] Ge H. (2023). Research on Intelligent Recognition Technology for Distance and Weather Type in Front of Explosive Transport Vehicles. Master’s Thesis.

[3] Wang B.J., Hensher D.A., Ton T. (2002). Safety in the road environment: A driver behavioural response perspective. Transportation.

[4] Jiang Q. (2023). Design and Implementation of a Video Surveillance Management System for Intelligent Identification of Urban Road Fire and Explosion Accidents. Master’s Thesis.

[5] Faisal M., Chaudhury S., Sankaran K.S., Raghavendra S., Chitra R.J., Eswaran M., Boddu R. (2022). Faster R-CNN Algorithm for Detection of Plastic Garbage in the Ocean: A Case for Turtle Preservation. Math. Probl. Eng..

[6] Fu H., Zhao H., Jiang J., Zhang Y., Liu G., Xiao W., Du S., Guo W., Liu X. (2024). Automatic detection tree crown and height using Mask R-CNN based on unmanned aerial vehicles images for biomass mapping. For. Ecol. Manag..

[7] Al-batat R., Angelopoulou A., Premkumar S., Hemanth J., Kapetanios E. (2022). An End-to-End Automated License Plate Recognition System Using YOLO Based Vehicle and License Plate Detection with Vehicle Classification. Sensors.

[8] Niu C., Song Y., Zhao X. (2023). SE-Lightweight YOLO: Higher Accuracy in YOLO Detection for Vehicle Inspection. Appl. Sci..

[9] Zhang X., Lei H., Yang S., Liu L., Shi Z., Yang G. (2023). Research on Workpiece Intelligent Detection Method Based on SSD Algorithm and Transfer Learning. Integr. Ferroelectr..

[10] Jiang T. (2023). Application of SSD network algorithm in panoramic video image vehicle detection system. Open Comput. Sci..

[11] Zhu Y., Zhe T., Liu Q. (2022). An Improved Faster R-CNN Vehicle Recognition Algorithm. Softw. Guide.

[12] Zhang Z., Yao G., Li X., Zhang J. (2021). Small target vehicle detection based on improved fast R-CNN algorithm. Technol. Innov. Appl..

[13] Zhou M. (2022). Pedestrian and vehicle detection algorithm based on YOLO model. Master’s Thesis.

[14] Yin Y., Xu Y., Xing Y. (2022). Vehicle Target Detection Algorithm Based on YOLO v4. Comput. Mod..

[15] Kang R. (2022). Research on Vehicle Target Detection Algorithm Based on SSD. Master’s Thesis.

[16] Yang F., Wu S. (2021). Research on Target Vehicle Detection Algorithm Based on SSD. IoT Technol..

[17] Hikvision—Leading the New Future of Intelligent IoT. https://www.hikvision.com/cn/.

[18] Su J., Liu Y. (2023). Vehicle Target Detection Algorithm Based on Improved YOLOv4. J. Tianjin Univ. Sci. Technol..

[19] Zhang P., Kang C., Tang W., Luo X. (2023). Real time detection of drone aerial video based on YOLOv4. J. Civ. Aviat. Flight Acad. China.

[20] Zhang X., Sun G., Lian M., Zhang Y. (2023). Fault Analysis of Power Supply Network in a Certain Unmanned System Based on Cellular Automata. Mod. Mach..

[21] Mou S. (2023). Research on Autonomous Lane Changing of Autonomous Vehicles Based on Game Theory. Master’s Thesis.

[22] Yu J. (2023). Research on Intelligent Vehicle Lane Changing with the Introduction of Game Theory. Master’s Thesis.

[23] Dong R. (2023). Research on Continuous Flow Cellular Automata Model for Narrow Motor Vehicle Lanes in Cities. Master’s Thesis.

